# “We Just Improvise”: Exploring Teachers’ Perspectives on Sport Participation for Learners with Intellectual Disabilities in Rural South Africa

**DOI:** 10.3390/ijerph22060893

**Published:** 2025-06-03

**Authors:** Avhasei Dorothy Meregi, Phumudzo Khangwelo Mulibana, Gudani Goodman Mukoma

**Affiliations:** Department of Biokinetics, Recreation and Sport Science, Faculty of Health Sciences, University of Venda, Thohoyandou 0950, South Africa; phumudzo.mulibana@univen.ac.za (P.K.M.); gudani.mukoma@univen.ac.za (G.G.M.)

**Keywords:** intellectual disability, school sport, inclusion, special education, Vhembe District, South Africa, physical activity, qualitative research

## Abstract

Background: Participation in sports offers children with intellectual disabilities (IDs) crucial opportunities for development. However, they often face barriers to inclusion in school-based sports, especially in under-resourced areas. This study aimed to (1) assess the level of participation in school sports among learners with IDs, and (2) explore teachers’ perceptions of the benefits and barriers to such participation in special schools within the Vhembe District of South Africa. Methods: A qualitative, descriptive research design was employed. Face-to-face semi-structured interviews were conducted with 20 teachers from four special schools. Thematic analysis helped identify key themes and interpret responses. Results: All schools offered weekly sports activities, as required by the Department of Education. Teachers viewed sports as vital for social interaction, physical fitness, and psychological well-being. However, barriers such as insufficient adapted equipment, inadequate facilities, and limited family support hindered meaningful participation, particularly for learners with profound disabilities. Conclusions: School sports have the potential to transform the lives of learners with IDs, but systemic barriers restrict access. Increased investment in inclusive infrastructure, adaptive equipment, teacher training, and community awareness is essential to align policy with practice in special education.

## 1. Introduction

Learners with intellectual disabilities (IDs) often face multiple layers of exclusion in education systems, particularly when it comes to participation in structured physical activity (PA) or sport, a widespread challenge [1,2]. Although sport offers well-documented benefits across physical, emotional, and social domains [3], learners with IDs remain significantly underrepresented in school-based sport initiatives [4]. These disparities are particularly concerning given that sport can serve as a powerful enabler of inclusion and development for learners with diverse abilities.

Sport participation has been shown to improve physical fitness, enhance social interaction, reduce stress, and promote psychological well-being in children generally [5,6,7]. For learners with IDs, it also supports motor skill development, self-confidence, and overall quality of life [1,8]. In school settings, where learners spend most of their day, sport programmes can offer regular, structured opportunities for PA and positive peer engagement [9,10]. Bailey [11,12] argues that school-based sport provides a regulated and safe environment where qualified educators can promote active lifestyles and healthy habits that may continue into adulthood.

Despite these benefits, participation rates in PA among children with intellectual disabilities remain low compared to their peers without disabilities [13,14]. Factors such as health disparities, environmental barriers, and limited access to inclusive programming contribute to this gap. The World Health Organisation (WHO) [15] and Pitchford et al. [16] have stressed the need for targeted interventions and inclusive health promotion programmes for people with IDs. However, much of the existing research remains focused on populations without disabilities, with limited exploration of how learners with IDs experience sport, particularly in rural, under-resourced contexts of the Global South.

In South Africa, inclusive education policy promotes equitable opportunities for all learners, including those with disabilities [17,18]. However, implementation is uneven, especially in rural areas, where schools contend with infrastructural challenges, resource shortages, and limited training [19,20]. Although the Department of Education mandates weekly physical activity sessions for special schools [21,22], little is known about how these programmes are experienced by teachers and learners or what factors support or hinder effective participation.

Recent national policy frameworks, such as the 2024 Women in Sport Policy Framework of the South African Department of Sport, Arts and Culture [23], along with the department’s 2020–2025 strategic plan [24], and the South African National Sport and Recreation plan [22], emphasise the importance of inclusive sport participation opportunities in schools for all learners, including those with disabilities. Yet, the implementation of these policies in rural special schools is constrained by resource limitations and systemic inequities.

Empirical studies from other Limpopo districts, such as Capricorn and Mopani, reveal similar challenges: inadequate facilities, untrained staff, and minimal community involvement in school sport [25,26]. Parallel issues have been documented in neighbouring countries, like Zimbabwe and Botswana, where inclusive initiatives are hindered by insufficient infrastructure and limited teacher preparation [27,28,29,30]. These findings point to a broader regional trend of underdeveloped inclusive sport practices in rural schools. However, few studies have explored how these dynamics specifically affect learners with intellectual disabilities in South Africa’s rural special schools. This gap is particularly pronounced in the Vhembe District, where socioeconomic and infrastructural challenges are compounded by limited empirical documentation.

To address these gaps, this study sought to investigate the level of participation in school sports among learners with IDs in Vhembe District special schools and explore teachers’ perceptions of the benefits and barriers that influence such participation.

This study was guided by the following research questions: What is the level of participation in school sports among learners with IDs in special schools within the Vhembe District? What do teachers perceive as the benefits of participating in school sports for learners with IDs? Additionally, what barriers do teachers identify that hinder participation in school sports for learners with IDs?

### Theoretical Framework

This study is guided by the Social Model of Disability, which asserts that disability results not from individual impairments, but from barriers embedded in social and physical environments [31,32,33]. This model contests deficit-based views that link limited participation to individuals’ impairments and instead emphasises the systemic issues, like inadequate accessible facilities, unsupportive infrastructure, and social marginalisation, that hinder the full participation of individuals with disabilities.

By utilising this framework, the research reframes the obstacles to sports participation as not unavoidable results of learners’ impairments but as alterable constraints produced and maintained by the education system, school practices, and broader socio-political frameworks. It also highlights the role of teachers as both facilitators and potential catalysts in developing inclusive practices. The Social Model is especially relevant in the South African context, where inclusive education policies are in place but frequently undermined by inconsistent implementation and resource disparities. Thus, the framework provides a critical lens through which to examine the transformative potential of sport and the institutional and structural barriers that must be addressed to realise inclusive participation for all learners.

## 2. Materials and Methods

### 2.1. Research Design

This study adopted a descriptive qualitative research design to explore teachers’ perspectives on the participation of learners with IDs in school sport. A qualitative approach was appropriate for gaining in-depth insights into teachers’ experiences, perceptions, and the contextual factors shaping sport participation in special schools.

### 2.2. Study Setting and Participants

The study was conducted in the Vhembe District of Limpopo Province, South Africa, which has four public special schools catering to learners with IDs. The total population across these schools included 49 teachers. To ensure anonymity and confidentiality, the schools were coded as School A (8 teachers), School B (14 teachers), School C (16 teachers), and School D (11 teachers).

Purposive sampling was used to ensure the inclusion of teachers (*n* = 20) with direct experience in both physical education and special education. Inclusion criteria included current teaching experience in special schools, direct involvement in delivering or supervising sports activities, and a background in special needs education. Teachers not directly involved in sport or physical education were excluded. This approach was intended to ensure that participants could provide informed, relevant insights into the delivery of physical activity programs in special education settings.

School principals assisted in identifying eligible teachers. The first five willing and eligible teachers per school were selected until the target sample of 20 was reached. Thematic saturation was monitored during coding and was considered reached when no new codes or themes emerged after the 18th interview. Specifically, all subthemes identified had been repeated across at least three different schools, with two final interviews confirming data redundancy.

### 2.3. Participant Demographics

The sample included (*n* = 9) men and (*n* = 11) women teachers, ranging in age from 29 to 58 years. Teaching experience in special education varied from 3 to over 20 years. All participants held formal teaching qualifications, and most had received in-service training related to inclusive or adaptive physical education. This diversity in experience and background provided a broad range of perspectives on learner participation in sport.

### 2.4. Data Collection

Data were collected using semi-structured face-to-face interviews, allowing for open-ended responses while ensuring participant consistency. The interview schedule included questions on the frequency and nature of learner participation in school sports, teachers’ perceptions of the benefits of participation, and perceived barriers to inclusion and engagement. Interviews were conducted in English by the primary researcher and audio-recorded with permission, and each lasted approximately 30–45 min. The open format also allowed for probing and clarification to deepen the understanding of participants’ responses. Interviews took place in quiet, private school spaces to ensure comfort and confidentiality. Field notes were also taken during and after each interview to capture non-verbal cues and contextual observations.

### 2.5. Pilot Testing and Instrument Validation

A pilot study was conducted in two special schools not part of the final sample. One participant was selected from each school to assess the clarity and relevance of the interview guide. Feedback from the pilot informed minor revisions to the wording and sequencing of questions. Additionally, the interview schedule was reviewed by two senior academics with expertise in inclusive education and disability studies to ensure conceptual clarity and alignment with research aims.

### 2.6. Ethical Considerations

Ethical approval for the study was granted by the University of Johannesburg (Ethics Clearance Number: REC-01-163-2016, approved on 20 September 2016). Further approval and access were obtained from the Limpopo Department of Education—Vhembe District Office and the principals of the participating schools. Written informed consent was obtained from all participants before data collection. The consent form included details on the study’s purpose, procedures, confidentiality, voluntary participation, and the right to withdraw at any stage without consequence.

### 2.7. Researcher Reflexivity

The researchers have a background in inclusive education and prior physical education teaching experience in special schools, contributing to an informed yet empathetic interview approach. Reflexive journaling was maintained throughout the research process to document potential biases, personal assumptions, and decisions during data collection and analysis. Peer debriefing and member checking were employed to enhance analytical objectivity and ensure that the findings authentically reflected participants’ views.

### 2.8. Data Analysis

All interviews were recorded in audio format, transcribed verbatim, and analysed using reflexive thematic analysis in accordance with Braun and Clarke’s six-phase approach [34]. This process involved gaining familiarity with the data through repeated reviews, manually generating initial codes, and organising these codes into meaningful categories. Next, overarching themes were identified within the coded information, followed by a review and refinement of these themes to ensure their accuracy and relevance. Finally, the themes were defined and labelled, with supporting excerpts from the data, culminating in a final report that integrates the analysis with the relevant literature and the study’s theoretical framework.

The first author conducted the coding manually using Microsoft Excel to track and organize codes across the dataset. This approach aligns with the critique of systemic exclusion found in the social model; supports the interpretive nature of qualitative inquiry; and respects the subjective, socially situated knowledge of teachers who work with learners with intellectual disabilities. Instead of relying on a rigid coding framework, such as axial or selective coding, the process was flexible and inductive, allowing themes to naturally emerge from the data. Themes were collaboratively developed through discussions among the research team to ensure coherence and alignment with the study’s research questions and theoretical framework (see Appendix B).

To ensure reliability, a second qualitative researcher independently reviewed a subset of the transcripts and codes. Any discrepancies were discussed and resolved through consensus. An audit trail was maintained throughout the process to document coding decisions and theme development.

### 2.9. Trustworthiness

To ensure the validity and reliability of the research findings, this study employed Lincoln and Guba’s [35] four criteria for trustworthiness in qualitative research: credibility, transferability, dependability, and confirmability.

Credibility was established through extensive interactions with participants, thorough examination of transcripts, and data triangulation by comparing cases across the four participating schools. The authenticity of the findings was further bolstered by peer debriefing and input from academic experts in disability and inclusive education. Detailed contextual descriptions of the research setting, participant demographics, and school environments supported transferability. This approach enables readers and researchers to evaluate the applicability of the findings in other contexts with similar characteristics. Dependability was addressed by maintaining a comprehensive audit trail of all research decisions, including the sampling methods, interview guides, transcription procedures, and data analysis steps. The research process was meticulously documented to ensure clarity and replicability. Confirmability was enhanced through reflective journaling and ongoing peer consultation, which helped mitigate potential researcher bias. All interpretations and conclusions were firmly rooted in the data, as evidenced by the inclusion of direct quotes from participants in the results.

## 3. Results

### 3.1. Participant Overview

The sample comprised 20 teachers selected evenly from four special schools (Schools A–D) within the Vhembe District, with each school contributing five participants. To ensure anonymity, teachers were assigned codes ranging from T1 to T20. The high response rate across all participating schools enabled comprehensive data collection, particularly in Schools B, C, and D, where every selected teacher engaged in the interviews. Among the 20 teachers, 7 (34%) reported holding a formal qualification in Physical Education (PE), while the remaining 13 (66%) did not possess any formal training in the subject. This indicates that a significant portion of the sample consisted of teachers lacking specialised qualifications in PE.

### 3.2. Participation in School Sports

All participants confirmed that learners with IDs at their schools engage in physical activity once a week, specifically on Wednesdays, as mandated by the Department of Education. This designated participation day is consistently observed across the district, providing a reliable routine for the learners. The sporting activities mentioned during the interviews include floor hockey, netball, athletics, bowling, catching, soccer, and football. Teachers emphasised that activities are not always designed to meet the diverse needs of learners with varying intellectual and physical abilities. They noted that students with mild-to-moderate intellectual disabilities can generally participate in group activities with some guidance. However, those with profound disabilities face significant challenges. These students often struggle to follow game rules, use equipment correctly, or engage in activities without one-on-one support. For instance, activities that require verbal instructions, complex coordination, or competitive gameplay are often inaccessible for many learners with profound disabilities, resulting in passive participation or complete exclusion. This situation underscores the need for adapted programs and individualised support strategies tailored to the severity of disabilities.

**Figure 1:**
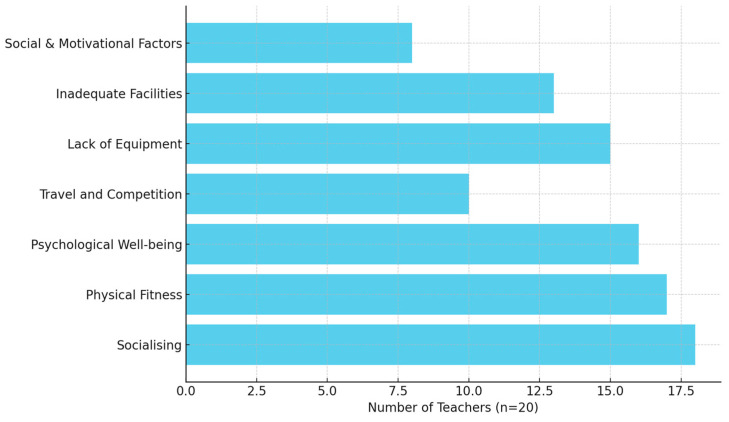
Frequency of mentions for each subtheme.

The thematic analysis conducted on teachers’ perspectives regarding sports participation for learners with IDs revealed two primary themes. The first theme, perceived benefits of sport participation, encompassed four subthemes: socialising and peer interaction; physical health and fitness; psychological and emotional well-being; and exposure, talent identification, and travel opportunities. The second theme addressed barriers to sport participation, which were categorised into three subthemes: lack of adapted equipment, inadequate facilities, and social and motivational factors. A summary of the overarching themes, their subthemes, and illustrative quotes from participants is presented in Table 1, with frequencies depicted in Figure 1. A detailed explanation of these themes follows below.

Figure 1 presents the frequency of mentions for each identified subtheme, providing a visual overview of which barriers and benefits were most salient across participants.

### 3.3. Theme 1: Perceived Benefits of Sport Participation

Teachers consistently emphasised that sports are valuable for promoting holistic development among learners with IDs. Under theme 1, four subthemes were identified, namely social engagement, physical fitness, psychological well-being, and exposure to travel and competition.

#### 3.3.1. Subtheme 1.1: Socialising and Peer Interaction

All respondents noted the role of sports in fostering interpersonal relationships and social skills. Learners who are typically withdrawn during classroom activities were seen to engage more actively during sports, highlighting the inclusive and socially liberating nature of physical play.

“*Sports helps in socialising; pupils play together, and they have fun. There are learners who don’t want to play with others, but when it comes to Wednesday, they all participate.*”

(T20)

This observation suggests that sports can act as a social equaliser, enabling even less communicative learners to find connection and shared experiences with peers.

#### 3.3.2. Subtheme 1.2: Physical Health and Fitness

All participants reported improvements in learners’ physical fitness due to regular sports activities. Teachers noted visible changes in energy levels, endurance, and muscle development.

“*It builds up their bodies, strong muscles, stimulates blood circulation. It makes learners to be active and it stimulates endurance.*”

(T1)

This aligns with the recognised role of physical activity in enhancing physical health in children with disabilities and highlights the potential of sports as a supplementary therapeutic tool.

#### 3.3.3. Subtheme 1.3: Psychological and Emotional Well-Being

Teachers observed that participation in sports had a calming and emotionally stabilising effect on learners. Sports were described as a coping mechanism for managing anxiety and stress.

“*sports releases tension and helps manage and cope with stress.*”

(T15)

The findings suggest that physical activity contributes positively to emotional regulation due to the structured nature of play, opportunities for success, and peer validation.

#### 3.3.4. Subtheme 1.4: Exposure, Talent Identification, and Travel Opportunities

Sports also provided a gateway for learners to travel beyond their school environment and engage in inter-school competitions. Teachers noted that these excursions often reveal previously unrecognised talents.

“*They benefit by going out to participate with others.*”

(T3)

This implies that structured sports programs offer essential experiential learning opportunities, exposing learners to broader social contexts and boosting self-esteem through recognition.

### 3.4. Theme 2: Barriers to Sport Participation

Despite teachers’ acknowledgement of the advantages of school sports, they also highlighted several ongoing barriers that prevent students with IDs from fully participating. Three subthemes emerged from discussions under theme 2, including the lack of adapted equipment, insufficient facilities, and social and motivational challenges.

#### 3.4.1. Subtheme 2.1: Lack of Adapted Equipment

A dominant concern across all schools was the unavailability of appropriate sports equipment. Teachers highlighted that existing resources were often inadequate, particularly for learners with more profound disabilities.

“*The school does not have equipment suitable for the sporting codes, especially equipment for profound learners.*”

(T10)

The mismatch between equipment and learners’ abilities results in exclusionary practices, even within structured programs. This underscores the need for targeted investment in adaptive equipment.

#### 3.4.2. Subtheme 2.2: Inadequate Facilities

Three out of four schools lacked dedicated sports facilities. Teachers reported that they often relied on open spaces or borrowed community fields to conduct activities. Only one school (School A) possessed a functional soccer field.

“*Our school has only one soccer field, for other activities, we just improvise on the available open space.*”

(T20)

This limitation affects sports offerings’ consistency, variety, and safety, especially during adverse weather or when community spaces are unavailable.

#### 3.4.3. Subtheme 2.3: Social and Motivational Factors

Teachers acknowledged that personal motivation, current skill levels, and support from peers and family shape some learners’ participation. While peer interaction was generally positive, family involvement was inconsistent.

“*You can see that those who can move around will always want to play with their friends, but they also need encouragement from their parents.*”

(T5)

The impact of these barriers varied depending on the severity of the disability. Learners with mild-to-moderate intellectual disabilities were more likely to participate in standard activities with minimal adaptations. However, they still faced challenges related to motivation and peer interactions. In contrast, learners with profound intellectual disabilities were significantly affected by the lack of adapted equipment and structured support. Their participation was often limited to observing, as they struggled with understanding instructions, using equipment, or managing overstimulation.

These findings highlight the need to tailor sports programs to accommodate the diverse functional abilities of all learners. They also reflect the relationship between the home environment and school engagement, emphasising the importance of community awareness and family education regarding the benefits of sports for learners with disabilities.

## 4. Limitations

The main limitation of the study is that the study covered only a small sample from Vhembe District, one district in Limpopo. The results of this study are based on information from teachers rather than from the learners themselves; this puts limits on the generalisation of the findings.

## 5. Discussion

This study set out to understand the level of participation in school sport among students with intellectual disabilities in special schools within the Vhembe District, while also investigating teachers’ perceptions of the benefits and barriers influencing this participation. The findings reveal a dual narrative; although sport is widely acknowledged as a vital instrument for holistic development, participation is frequently restricted by infrastructural, social, and systemic challenges.

Teachers consistently highlighted the multifaceted benefits of sports for learners with IDs, aligning with previous studies emphasising the role of physical activity in promoting inclusion, well-being, and development [36,37,38]. Sport was described as an inclusive space where even socially withdrawn learners engaged with peers, suggesting that physical activity serves as a non-verbal medium for communication and friendship building, particularly critical for learners with expressive or receptive language challenges.

The emphasis on psychological well-being further supports research indicating that regular participation in adapted physical activity helps mitigate anxiety and improve mood [39,40]. In resource-constrained environments, such psychosocial gains may be even more significant, offering learners rare opportunities to express themselves freely and experience personal achievement outside academic settings. Moreover, the exposure to travel and competition provided through sports outings fosters motivation and talent recognition. This resonates with Burnett’s [41] notion of “sport-for-development” in under-resourced areas, where sports become a mechanism for broader social and educational gains.

Despite the clear benefits, the findings highlight several barriers that hinder sustained and inclusive participation. One of the main challenges is the lack of appropriate equipment and sports facilities [1,42]. Teachers have consistently reported that the existing equipment does not meet the needs of learners with profound intellectual or multiple disabilities. This situation reflects broader inequalities in South African special education settings, where underfunding and infrastructure gaps often impede effective program delivery [43,44].

In addition to material shortages, contextual improvisation, such as using unstructured open spaces for sports, limits the scope of activities and may pose safety risks. While indicative of teacher dedication, such improvisation signals the need for systematic policy support and investment in inclusive sports infrastructure at the district and provincial levels.

On the social front, peer and parental influences shaped learners’ participation. This finding mirrors prior research suggesting that learner motivation is individually driven and significantly affected by familial and social reinforcement [37,45]. Where families are disengaged or unaware of the value of sport for learners with disabilities, participation often declines. This points to the need for parental sensitisation programs to foster consistent encouragement and collaboration between school and home.

While the Department of Education’s provision for Wednesday sports suggests formal inclusion of learners with IDs in physical education, the real-world implementation often falls short. The once-a-week schedule, lack of adapted resources, and low community integration illustrate a gap between policy intent and lived practice. Without dedicated support, learners risk being symbolically included but practically excluded, a common tension in disability-inclusive education [44,46].

Active participation in sports has a significant role to play in the holistic development of learners with IDs, and the results indicated that learners with IDs do participate in sports at school. Organised sports activities are conducted on Wednesdays. It was noted that learners also participate in Special Olympics programs and other competitions.

These findings have several implications. To realise the full potential of school sport for learners with IDs, a multi-level approach is needed. At the school level, staff need access to adaptive equipment and in-service training in inclusive sports facilitation. At the community level, partnerships with local clubs or sports hubs could widen learners’ exposure and support. At the policy level, funding allocations must reflect the material needs of special schools, with routine monitoring of implementation effectiveness. Furthermore, efforts should be made to amplify the learner voice in the design of school sports programs, ensuring that sports are both developmentally appropriate and personally meaningful.

## 6. Strengths and Limitations

While this study offers valuable insights into teachers’ perspectives on the inclusion of learners with IDs in school sport, several limitations should be acknowledged. First, the sample was limited to 20 teachers from one district in Limpopo Province, which may affect the generalisability of the findings to other regions or school contexts. Although data saturation was achieved, the perspectives captured reflect those of teachers willing and available to participate, potentially excluding the voices of those with differing experiences. Additionally, the reliance on self-reported data through interviews may introduce social desirability bias, where participants respond in a way they believe is expected. Despite these limitations, methodological rigour through triangulation, member checking, and reflexive practices was applied to ensure the trustworthiness of the findings.

## 7. Conclusions

This study explored teachers’ perspectives on the participation of learners with intellectual disabilities in school sport across four special schools in the Vhembe District of South Africa. The findings confirm that sport empowers social connection, emotional well-being, physical development, and inclusion. Teachers observed that even learners who are less engaged academically tend to thrive during sports sessions, highlighting the unique value of PA in the developmental journey of learners with IDs.

However, these benefits are tempered by persistent barriers, including a lack of adapted sports equipment, inadequate facilities, and limited family engagement. The gap between inclusive policy frameworks and their implementation reveals a tension between symbolic inclusion and practical exclusion, where learners are formally included in school sport but often unable to participate meaningfully. Based on the frequency of mentions during the interviews, several key priorities emerge. First, the provision of adapted equipment is recognised as a significant constraint by 15 out of 20 participants. Second, 13 out of 20 participants express concern over the need for upgraded facilities, citing inadequate fields and safe play spaces. Third, 12 out of 20 participants emphasise the importance of teacher training in inclusive sports, indicating a crucial need for enhanced guidance. Lastly, 8 out of 20 participants stressed the necessity of parental engagement campaigns, highlighting the lack of support from home.

To move from tokenism toward genuine inclusion, coordinated efforts are needed across school, community, and policy levels to invest in accessible infrastructure, teacher training, and parental awareness. These changes will ensure that sports become not only a weekly routine but a transformative, inclusive space for all learners, regardless of ability.

## Figures and Tables

**Table 1:** Summary of thematic analysis of teachers’ perspectives of sports participation for learners with intellectual disabilities.

Theme	Subtheme	Description	Illustrative Quote
1. Benefits of sport participation	1.1. Socialising	Sport promotes interaction and inclusion, encouraging learners to form friendships and socialise.	“…when it comes to Wednesday, they all participate.” (T20)
	1.2. Physical fitness	Physical activity improves strength, circulation, and endurance.	“It builds up their bodies…stimulates endurance.” (T1)
	1.3. Psychological well-being	Sport reduces stress and contributes to emotional stability.	“Sports releases tension and helps manage stress.” (T15)
	1.4. Travel and competition opportunities	Learners travel and participate in competitions, enabling skill discovery and exposure to new environments.	“They benefit by going out to participate with others.” (T3)
2. Barriers to sport participation	2.1. Lack of equipment	Inadequate or non-adaptive equipment limits learner engagement.	“…not suitable for profound learners.” (T10)
	2.2. Inadequate facilities	Schools lack proper sports infrastructure, relying on improvised or shared spaces.	“We just improvise on the available open space.” (T20)
	2.3 Social and motivational factors	Participation varies based on learner ability, motivation, peer interaction, and parental support.	“They need encouragement from their parents.” (T5)

## Data Availability

The data generated and analysed during this study are available upon reasonable request from the corresponding author. Due to the sensitive nature of the data, requests will be reviewed, and access will be granted in accordance with ethical guidelines and participant consent.

## Abbreviations

The following abbreviations are used in this manuscript:

ID	intellectual disability
PA	physical activity
WHO	World Health Organisation
ADM	Avhasei Dorothy Meregi
PKM	Phumudzo Khangwelo Mulibana
GGM	Gudani Goodman Mukoma

## Appendix A. Semi-Structured Interview Guide for Exploring Teachers’ Perspectives on Sport Participation for Learners with Intellectual Disabilities in Rural South Africa

Purpose of Interview:

To explore school principals’ perspectives on the level of participation, available resources, perceived benefits, and barriers related to school-based physical activity (PA) programs for learners with intellectual disabilities.

Section A: Introduction and Rapport Building.

1. Thank you for taking the time to meet with me today. To begin, can you briefly tell me about your school and your role as principal?

Section B: Physical Activity Programs at the School.

2. Availability of Programs
  - Does your school offer any physical activity (PA) programs for learners?
  - If yes, what kinds of physical activities are included? (e.g., sports, movement games, and exercise sessions).
3. Facilities and Equipment
  - What facilities and equipment are available to support these physical activities?
  - Are these facilities adapted for learners with different types of intellectual disabilities?
4. Stakeholders Involved
  - Who are the key stakeholders involved in organising or supporting these activities?
  - (Prompt: NGOs, Department of Education, parents, and external coaches).

Section C: Participation and Inclusion.

5. Learner Participation
  - Do you know how many learners actively participate in these activities?
  - How often do these activities take place (daily, weekly)?
  - Is participation optional or mandatory?
6. Competitions and Events
  - Do learners participate in any form of competition?
  - Can you describe the kinds of events or competitions they are involved in? (Prompt: inter-school sports and district events).
7. Non-Participation
  - For those who do not participate, what do you think are the main reasons?
  - (Prompt: physical limitations, lack of interest, transportation, and parental support).
8. Lunch Break Activities
  - What do learners typically do during lunch breaks?
  - Are they physically active during this time?

Section D: Perceptions and Role of School Leadership.

9. Perceived Benefits of Participation
  - What are your thoughts on the benefits of physical activity for learners with intellectual disabilities?
10. Role of the Principal
  - Do you see yourself as playing a role in encouraging learners to be physically active?
  - If so, how do you do this?

Section E: Barriers and Support Needs.

11. Challenges and Barriers
  1. What are your school’s main challenges in implementing physical activity programs for learners with intellectual disabilities?
12. Support and Improvement
  1. What support do you think schools need to improve participation and inclusion in physical activities?
  2. (Prompt: funding, infrastructure, staff training, and equipment).

Section F: Closing.

13. Is there anything else you would like to share about physical activity at your school or any recommendations for improving participation?

## Appendix B. Sample Coding Matrix and Analytical Memos

**Table A1:** Example of initial codes grouped into themes.

Initial Code	Subtheme	Main Theme	Illustrative Quote
“No equipment for profound learners”	Lack of adapted equipment	Barriers to sport participation	“We don’t have proper equipment for profound learners” (T10).
“We use open space, sometimes even under trees”	Inadequate facilities	Barriers to sport participation	“We use open spaces, even under trees sometimes” (T20).
“Parents don’t always support”	Social and motivational challenges	Barriers to sport participation	“They need encouragement from their parents” (T5).
“They play and laugh together”	Socialising and peer interaction	Benefits of sport participation	“They all come together during sports” (T20).
“They’re stronger now”	Physical health and fitness	Benefits of sport participation	“It makes them strong and active” (T1).
“Sports helps reduce stress”	Psychological and emotional well-being	Benefits of sport participation	“It helps release their stress” (T15).
“Learners travel for competition”	Travel and exposure opportunities	Benefits of sport participation	“They travel and show what they can do” (T3).

**Table A2:** Excerpts from analytical memos.

Date	Memo Title	Summary/Insight
12 February 2024	“Social Impact of Sport”	Several teachers highlighted how sports activities enhance peer relationships for typically withdrawn learners.
15 February 2024	“Profound Disabilities and Exclusion”	Equipment and instruction not adapted to learners with profound IDs, leading to passive or no participation.
17 February 2024	“Parental Role”	Emergent pattern of minimal parental involvement across schools; teachers feel this limits learner engagement.
20 February 2024	“Saturation Achieved”	By the 18th interview, no new themes or codes were emerging, confirming data saturation for this sample.
